# Risk Analysis of 24 Residual Antibiotics in Poultry Eggs in Shandong, China (2018–2020)

**DOI:** 10.3390/vetsci9030126

**Published:** 2022-03-10

**Authors:** Xiaoyu Ma, Ling Chen, Lingling Yin, Youzhi Li, Xiuzhen Yang, Zhiguo Yang, Guihua Li, Hu Shan

**Affiliations:** 1 College of Veterinary Medicine, Qingdao Agricultural University, Qingdao 266109, China; 2 Shandong Center for Quality Control of Feed and Veterinary Drugs, Jinan 250010, China; 13708935340@163.com (L.C.); sdlinglingyin@126.com (L.Y.); liyouzhi2009@126.com (Y.L.); 3 Shandong Provincial Center for Quality and Safety of Animal Products, Jinan 250010, China; yangxiuzhenaaa@126.com (X.Y.); sdfeedsyang@163.com (Z.Y.); guihua69@163.com (G.L.)

**Keywords:** Shandong, antibiotic residues, IFS, risk evaluation

## Abstract

Although antibiotics have played a certain positive role in the prevention and treatment of poultry diseases, as well as the promotion of poultry growth, some farmers use antibiotics in an incorrect way in the breeding process, resulting in antibiotic residues in poultry tissues, organs and edible products. Residual antibiotics enter the human body through the food chain and accumulate, which not only causes poisoning and allergic reactions, but also drug resistance of pathogenic microorganisms, thus endangering the health of consumers. In this investigation, the residues of 24 antibiotics, including fluoroquinolones, sulfonamides, macrolides, tetracyclines, antivirals, lincomycin and florfenicol, were analyzed in 1211 poultry egg samples in Shandong, China, from 2018 to 2020. Then, based on the per capita intake of poultry eggs recommended in the dietary guidelines of Chinese residents, the maximum residue limit of veterinary drugs specified in Chinese regulations and the average weight of males and females aged 18 and over in 2020, the risk of residual antibiotics was evaluated by International Food Safety indices (IFS). The detection results showed that 104 of 1211 samples were detected with antibiotic residues, with a detection rate of 8.58%. Among them, the main residues were enrofloxacin, sulfonamides and florfenicol. The IFS calculation results showed that the IFS of residual antibiotics ranged from 1.44 × 10^−7^ to 0.102. Therefore, although enrofloxacin, sarafloxacin, danofloxacin, sulfonamides, tilmicosin, doxycycline, florfenicol, which are banned during egg laying, were detected in poultry eggs in Shandong, these residues did not pose a threat to the health of Chinese adult consumers, according to the daily dietary habits of Chinese people. However, it is strongly suggested that Shandong should strengthen the monitoring of antibiotic use during egg laying.

## 1. Introduction

It is understood that the quantity and variety of antibiotics used in the breeding industry in China are at the forefront of the world, and the total number of antibiotics used in 2018 alone reached 29,774.09 tons [1]. Antibiotics are widely used in China’s poultry industry. Many farmers tend to use antibiotics as feed supplements in poultry rations to improve growth and prevent disease infection [2]. However, if antibiotics are not used rationally, such as over-dose use, long-term use of low dose and unreasonable mixing [3,4], it may lead to antibiotic residues in products. Residual drugs harm residents’ health in many aspects, such as causing the occurrence of “mutagenic, teratogenic, carcinogenic” toxic actions [5], inducing drug-resistant strains [6], leading to gastrointestinal disorders [7,8], toxic reactions [9], allergic reactions [10], etc. Various countries and relevant international organizations have established regulations to control antibiotic residues in animal products. For example, both the United States and the European Union have issued regulations to set maximum residue limits for antibiotics in poultry products [11,12]. China also issued the National Food Safety Standard Maximum Residue Limits for Veterinary Drugs in Food in 2019 [13], which stipulates the maximum daily intake of poultry antibiotics and the antibiotics prohibited during egg laying.

Even so, unreasonable residues of antibiotics have been detected frequently in poultry eggs in many countries [14,15,16,17]. This not only challenges the quality and safety supervision of poultry products, but also seriously affects people’s consumer confidence in poultry products. Accordingly, people apply risk evaluation to evaluate the safety of food [18]. Food risk evaluation experts from the Codex Alimentarius Commission (CAC) and the World Health Organization (WHO) constructed the International Food Safety indices (IFS) as an evaluation method of food safety risk by using the quantitative relationship between the actual and safe intake of hazardous substances [19].

By analyzing the evaluation results, we can effectively identify the degree of harm of risk factors in food to consumers’ health [20]. Shandong is a big producer and consumer of poultry eggs, and its eggs output has been at the forefront of China for several consecutive years. Therefore, in this study, 24 antibiotics with high risk in Shandong poultry breeding were firstly selected as detection indicators. Secondly, antibiotic residues were determined to detect the presence of antibiotic residues in eggs collected from poultry farms, supermarkets and farmers’ markets in 16 cities in Shandong, China. Finally, IFS was used to evaluate the safety of residual antibiotics. This study provides the latest information on antibiotic residues in poultry eggs, which is of great significance to ensure consumer health.

## 2. Materials and Methods

### 2.1. Sampling

A total of 1211 eggs were collected from poultry farms, supermarkets and farmers’ markets in 16 cities in Shandong, China, from 2018 to 2020. Of these, 501 were collected in 2018, 350 in 2019 and 360 in 2020.

All samples were transported to laboratory on ice within 12 h of collection and immediately refrigerated upon arrival.

### 2.2. Antibiotic Residues Testing

The concentrations of 24 antibiotics in egg liquid were determined by ultra-performance liquid chromatography–tandem mass spectrometry (Waters xevo TQ-S, Shanghai woteshi Technology Co., Ltd., Shanghai, China). This method was described and verified in detail in File S1 (Extraction and analytical method, validation process for the analysis of the eggs in the UPLC-MS spectrometry). The tested antibiotics included Fluoroquinolones (Enrofloxacin, Sarafloxacin, Darfloxacin, Norfloxacin, Lomefloxacin), Sulfonamides (Sulfamonomethoxine, Sulfadimidine, Sulfamethoxazole, Sulfadimethoxine, Sulfaquinoxaline, Sulfachloropyrazine sodium, Sulfaclodazine sodium), Antivirals (Amantadine, Rimantadine), Macrolides (Tylosin, Tilmicosin, Erythromycin, Azithromycin), Tetracyclines (Oxytetracycline, Tetracycline, Doxycycline, Aureomycin), Lincomycin and Florfenicol.

#### 2.2.1. Extraction of Antibiotics

First, 2.00 ± 0.02 g of mixed egg liquid was weighed into a 50 mL centrifuge tube and spiked with 2 mL of 0.02 mol/L EDTA solution. After 1 minute of vortexing, 8 mL of acetonitrile was added to the solution. Then, the solution was subjected to vortices for 1 min, ultrasonic extraction for 10 min and high-speed centrifugation at 5000× *g* r/min for 5 min. Without activation and balance, 5 mL of supernatant from the treated solution was directly loaded into a 6cc PRiME HLB solid-phase extraction column, and all effluent from the column was collected. After the effluent was blown to nearly dry with nitrogen at 40 °C, it was constant volume at 1.00 mL with 20% methanol aqueous solution. Finally, the solution was filtered with 0.2 μm microporous membrane.

#### 2.2.2. Detection of Antibiotics

Chromatographic column: BEH C18 (100 mm × 2.1 mm, 1.7 μm); Flow rate: 0.2 mL/min; Injection volume: 1 μL; Column temperature: 35 °C.

Mobile phase: in positive ion mode, mobile phase A is methanol, and mobile phase B is 0.1% formic acid water; in negative ion mode, mobile phase A is methanol, and mobile phase B is water.

In this study, the processing principles of undetected data are as follows: when more than 60% data are undetected, undetectable data are replaced for “Limit of Detection (LOD)”. When 60% and less data are undetected, undetectable data are replaced for 1/2 LOD. Table 1 shows the limit of detection (LOD) of 24 antibiotics.

### 2.3. Risk Evaluation of Antibiotic Residues in Poultry Eggs

According to the detection results, International Food Safety indices (IFS) were used to evaluate the antibiotic residues in the samples. 

The evaluation formula is

IFS = (R ∗ F)/(SI ∗ *bw*)

R is the concentration of antibiotic residue in poultry eggs, expressed as μg/kg.

F is daily consumption of poultry eggs, expressed as kg/person/d. We referred to the recommended intake in the Chinese Dietary Guidelines (2019) [21] and selected 25 g/person/d and 50 g/person/d.

SI is the allowable daily intake (ADI) of antibiotics, expressed as μg/kg *bw*. This study was based on the provisions of the National Food Safety Standard Maximum Residue Limits for Veterinary Drugs in Food [13]; 

*bw* is the average body weight, expressed as kg. According to the report on Nutrition and Chronic Diseases of Chinese Residents (2020), the average body weight of males aged 18 and over was 69.6 kg, and that of females was 59 kg.

When IFS < 0.01, it is essentially harmless to consumer health. When IFS < 1, the harm to consumer health is acceptable. When IFS > 1, the risk to consumer health is unacceptable.

## 3. Analysis and Results

### 3.1. Residues of 24 Antibiotics in Poultry Eggs

Antibiotic residues were detected in 104 of 1211 eggs detected by HPLC/MS (Table 2), with a detection rate of 8.85%. A total of 12 antibiotics were detected, including enrofloxacin, sarafloxacin, darfloxacin, sulfamonomethoxine, sulfadimidine, sulfamethoxazole, sulfaquinoxaline, sulfachloropyrazine sodium, tilmicosin, doxycycline, lincomycin and florfenicol (Table 3, Table 4 and Table 5). According to the regulations of China’s Ministry of Agriculture, lincomycin is an antibiotic restricted to egg laying, and the other 11 antibiotics are prohibited for egg laying. The results also apply to the EU and CAC.

In the detection of fluoroquinolones, enrofloxacin was detected in three consecutive years, with one in 2018, four in 2019 and three in 2020 (Table 3, Table 4 and Table 5). In this investigation, the highest residual concentration of enrofloxacin reached 193.08 μg/kg in 2020. Only two and one of sarafloxacin and darfloxacin were detected in 2020 (Table 5). The number of sarafloxacin and darfloxacin undetected exceeded 90%, so data were processed with LOD of 0.5 and 0.1 μg/kg. Combined with the results of HPLC/MS, the average residual concentrations of sarafloxacin and darfloxacin were lower than 1 μg/kg, respectively.

Seven kinds of commonly used sulfonamides were used as detection indices, and five kinds were detected. Table 3, Table 4 and Table 5 clearly show that antibiotic residues were detected in 19, 11 and 9 eggs per year, respectively. Among them, sulfamonomethoxine and sulfadimethoxine were detected for three consecutive years. During these three years, the sulfonamides detected in eggs showed irregularity, which may be related to illegal drug use during egg laying.

Tilmicosin was the only macrolide detected, with only four in 2018 (Table 3).

As the only tetracycline detected, doxycycline was detected in a small numbers (five and two) in 2018 and 2020 (Table 3 and Table 5).

Florfenicol was the most detected antibiotic in this survey. In 2019, florfenicol residues were found in as many as 26 eggs (Table 4). The residual concentration of florfenicol ranged from 0.02 to 360.69 μg/kg. As a banned antibiotic during laying, its test results were not encouraging.

Lincomycin was the only one of the 11 antibiotics that can be used in laying hens. The detected numbers were one in 2018 and three in 2020 (Table 3 and Table 5). Although lincomycin is listed as a restricted antibiotic for egg laying, China does not set a maximum residue limit in eggs.

No residues of norfloxacin, lomefloxacin, sulfadimethoxine, sulfaclodazine sodium, amantadine, rimantadine, tylosinamantadine, erythromycin, azithromycin, oxytetracycline and aureomycin were detected from 2018 to 2020.

### 3.2. Risk Evaluation of Poultry Eggs in Shandong

The risk of antibiotic residues was evaluated according to HPLC/MS results. In the calculation, 25 g/person/d and 50 g/person/d were selected to calculate the IFS of minimum antibiotic residues, maximum antibiotic residues and average antibiotic residues, respectively (Table 6 and Table 7).

The results showed that IFS ranged from 1.44 × 10^−7^ to 5.09 × 10^−2^ when Chinese adults ingested 25 g of eggs per day. IFS ranged from 1.44 × 10^−7^ to 0.102 when Chinese adults ingested 50 g of poultry eggs per day. According to IFS evaluation criteria, IFS is less than 0.1, indicating that the risk of residues is harmless to consumers.

## 4. Discussion

The residue of antibiotics is very important for food quality and safety. Enrofloxacin, as a synthetic and low-cost broad-spectrum antibiotic, has a good curative effect in the prevention and treatment of fowl typhoid, mycoplasma gallisepticum infection and pullorosis. Therefore, enrofloxacin is widely used in poultry breeding, but it is prohibited for use during egg laying. However, cases of enrofloxacin detected in poultry eggs still occur from time to time [22,23,24]. Enrofloxacin was detected in this investigation for three consecutive years, which is undoubtedly inconsistent with the regulations. Although the sample size of poultry eggs detected in this study was small and could not represent the pollution level of enrofloxacin residues in the whole Shandong province, it can also reflect the fact antibiotics were used in the off-duty period in the breeding process to a certain extent. This should attract Shandong’s attention.

Previous studies have shown that sulfonamides can compete for dihydrofolate synthase competitively, thus affecting the formation of nucleic acid to inhibit the growth and reproduction of bacteria [25]. In addition, they can inhibit some parasites, such as plasmodium and amoeba [26,27]. This good prevention and treatment effect of sulfonamides is widely welcomed by farms. However, China’s Ministry of Agriculture has banned the use of sulfonamides in laying hens. This investigation found that sulfonamides residues were detected in the eggs from some farms in Shandong. However, except for a few large concentrations, the residues of most detected sulfonamides were less than 10 μg/kg. According to previous studies, sulfonamides can still present residues in eggs 10–30 days after drug withdrawal [28,29]. Since the random sampling principle was adopted in this survey, the egg-laying period was not distinguished in detail, which may be one of the reasons for the detection of sulfonamides.

Residues of temicorin, doxycycline and lincomycin were also detected. However, they were not detected every year, and the detected quantity and residual concentration were not high, so it could not explain the overall residual pollution situation of Shandong farms.

Florfenicol is a broad-spectrum antibiotic. In China, florfenicol is forbidden during egg laying, but it can be used as a preventive medicine during growing. Studies have shown that florfenicol has stable chemical properties and long half-life. Poultry often need more than 10 days to eliminate the effects of drugs [30]. Therefore, if it is used in the later stage of growing, it would easily cause residues in eggs. This may be one of the reasons for the detection of florfenicol residues in Shandong for three consecutive years. However, it is undeniable that this is not the main reason for the detection of florfenicol. Shandong farms still have a great probability of illegal use of florfenicol.

Food safety evaluation can help people judge the impact of hazards on consumers’ health. The IFS results of this survey show that the residues of these illegally added antibiotics were harmless to the health of Chinese adult consumers. Although China’s Ministry of Agriculture has strict regulations on medication and daily allowable intake during egg laying, in fact, they also fully consider the elderly, children and sensitive people when formulating ADI. If only adult consumers are considered, ADI should increase the safety factor by 100 times, that is, ADI = non-toxic amount/100. Therefore, the residual concentration detected in this study would be very small if reduced by this ratio.

## 5. Conclusions

In this study, the residues of 12 antibiotics, such as enrofloxacin, sarafloxacin, darfloxacin, sulfamonomethoxine, sulfadimidine, sulfamethoxazole, sulfaquinoxaline, sulfachloropyrazine sodium, tilmicosin, doxycycline, lincomycin and florfenicol were detected in 104 of 1211 poultry eggs. According to the IFS risk evaluation, these residues have no impact on the health of adult consumers if they follow the egg intake habits of Chinese consumers (25–50 g egg intake per day). However, Shandong still needs to strengthen the supervision of these 12 antibiotics, especially enrofloxacin, sulfonamides and florfenicol.

## Figures and Tables

**Table 1:** Limit of detection (LOD) of 24 antibiotics.

Antibiotcs	Limit of Detection (μg/kg)
Enrofloxacin	0.1
Sarafloxacin	0.5
Darfloxacin	0.1
Norfloxacin	0.5
Lomefloxacin	0.02
Sulfamonomethoxine	0.02
Sulfadimidine	0.02
Sulfamethoxazole	0.1
Sulfadimethoxine	0.02
Sulfaquinoxaline	0.2
Sulfachloropyrazine sodium	0.2
Sulfaclodazine sodium	0.02
Amantadine	0.5
Rimantadine	0.5
Tylosin	0.2
Tilmicosin	0.5
Erythromycin	0.02
Azithromycin	0.5
Oxytetracycline	0.02
Tetracycline	0.02
Doxycycline	0.02
Aureomycin	0.2
Lincomycin	0.02
Florfenicol	0.02

**Table 2:** Detection of antibiotics from 2018 to 2020.

Years	Detection Rate %	Average Detection Rate %
2018	8.98 (45/501)	8.59% (104/1211)
2019	10.86 (38/350)	
2020	5.83 (21/360)	

**Table 3:** Residues of antibiotics in eggs in 2018.

Antibiotics		Detectable Numbers	Detectable Rate %	Average Detectable Numbers	Average Detectable Rate %	Residual Concentration (μg/kg)		
						Min	Max	Average
Fluoroquin-olones	Enrofloxacin	1	0.20	1	0.20	ND	81.58	0.2631
	Sarafloxacin	ND	ND			ND	ND	ND
	Darfloxacin	ND	ND			ND	ND	ND
	Pefloxacin	ND	ND			ND	ND	ND
	Lomefloxacin	ND	ND			ND	ND	ND
Sulfonamid-es	Sulfamonometho-xine	4	0.80	19	3.40	ND	1.18	0.0254
	Sulfadimidine	2	0.40			ND	5.89	0.0285
	Sulfamethoxazole	4	0.80			ND	91.26	0.3035
	Sulfadimethoxine	ND	ND			ND	ND	ND
	Sulfaquinoxaline	6	1.20			ND	13.11	0.2460
	Sulfachloropyrazi-ne sodium	3	0.60			ND	224	0.5625
	Sulfaclodazine sodium	ND	ND			ND	ND	ND
Antivirals	Amantadine	ND	ND	ND	ND	ND	ND	ND
	Rimantadine	ND	ND			ND	ND	ND
Macrolides	Tylosin	ND	ND	4	0.80	ND	ND	ND
	Tilmicosin	4	0.80			ND	24.21	0.5739
	Erythromycin	ND	ND			ND	ND	ND
	Azithromycin	ND	ND			ND	ND	ND
Tetracyclin-es	Oxytetracycline	ND	ND	5	1.00	ND	ND	ND
	Tetracycline	ND	ND			ND	ND	ND
	Doxycycline	5	1.00			ND	22.31	0.0539
	Aureomycin	ND	ND			ND	ND	ND
Other	Lincomycin	1	0.20	1	0.20	ND	8.05	0.0360
	Florfenicol	15	3.00	15	3.00	ND	106.8	2.2990

Note: ND means not detected.

**Table 4:** Residues of antibiotics in eggs in 2019.

Antibiotics		Detectable Numbers	Detectable Rate %	Average Detectable Numbers	Average Detectable Rate %	Residual Concentration (μg/kg)		
						Min	Max	Average
Fluoroquino-lones	Enrofloxacin	4	1.14	4	1.14	ND	1.94	0.1143
	Sarafloxacin	ND	ND			ND	ND	ND
	Darfloxacin	ND	ND			ND	ND	ND
	Pefloxacin	ND	ND			ND	ND	ND
	Lomefloxacin	ND	ND			ND	ND	ND
Sulfonamid-es	Sulfamonometho-xine	5	1.43	11	3.14	ND	99.07	0.5601
	Sulfadimidine	1	0.29			ND	7.44	0.0412
	Sulfamethoxazole	2	0.57			ND	7.30	0.1116
	Sulfadimethoxine	ND	ND			ND	ND	ND
	Sulfaquinoxaline	3	0.86			ND	4.20	0.2240
	Sulfachloropyraz-ine sodium	ND	ND			ND	ND	ND
	Sulfaclodazine sodium	ND	ND			ND	ND	ND
Antivirals	Amantadine	ND	ND	ND	ND	ND	ND	ND
	Rimantadine	ND	ND			ND	ND	ND
Macrolides	Tylosin	ND	ND	ND	ND	ND	ND	ND
	Tilmicosin	ND	ND			ND	ND	ND
	Erythromycin	ND	ND			ND	ND	ND
	Azithromycin	ND	ND			ND	ND	ND
Tetracyclines	Oxytetracycline	ND	ND	ND	ND	ND	ND	ND
	Tetracycline	ND	ND			ND	ND	ND
	Doxycycline	ND	ND			ND	ND	ND
	Aureomycin	ND	ND			ND	ND	ND
Other	Lincomycin	ND	ND	ND	ND	ND	ND	ND
	Florfenicol	26	7.43	26	7.43	ND	290.79	1.011

**Table 5:** Residues of antibiotics in eggs in 2020.

Antibiotics		Detectable Numbers	Detectable Rate %	Average Detectable Numbers	Average Detectable Rate %	Residual Concentration (μg/kg)		
						Min	Max	Average
Fluoroquino-lones	Enrofloxacin	3	0.83	6	1.67	ND	193.08	0.7169
	Sarafloxacin	2	0.56			ND	12.51	0.5333
	Darfloxacin	1	0.28			ND	2.13	0.1056
	Pefloxacin	ND	ND			ND	ND	ND
	Lomefloxacin	ND	ND			ND	ND	ND
Sulfonamid-es	Sulfamonometho-xine	3	0.83	9	2.5	ND	350.88	0.5194
	Sulfadimidine	ND	ND			ND	ND	ND
	Sulfamethoxazole	ND	ND			ND	ND	ND
	Sulfadimethoxine	ND	ND			ND	ND	ND
	Sulfaquinoxaline	2	0.56			ND	2.24	0.2049
	Sulfachloropyraz-ine sodium	4	1.11			ND	6.23	0.2278
	Sulfaclodazine sodium	ND	ND			ND	ND	ND
Antivirals	Amantadine	ND	ND	ND	ND	ND	ND	ND
	Rimantadine	ND	ND			ND	ND	
Macrolides	Tylosin	ND	ND	ND	ND	ND	ND	ND
	Tilmicosin	ND	ND			ND	ND	ND
	Erythromycin	ND	ND			ND	ND	ND
	Azithromycin	ND	ND			ND	ND	ND
Tetracyclines	Oxytetracycline	ND	ND	2	0.56	ND	ND	ND
	Tetracycline	ND	ND			ND	ND	ND
	Doxycycline	2	0.56			ND	24.29	0.1194
	Aureomycin	ND	ND			ND	ND	ND
Other	Lincomycin	3	0.83	3	0.83	ND	12.21	0.0766
	Florfenicol	7	1.94	7	1.94	ND	360.69	0.9957

**Table 6:** International Food Safety indices (IFS) of poultry eggs in Shandong, 2018–2020 (average intake of eggs, 25 g/d/person).

Antibiotics		Concentration (μg/kg)	2018		2019		2020	
			Male	Female	Male	Female	Male	Female
Fluoroquinolones	Enrofloxacin	Min	5.79 × 10^−5^	6.83 × 10^−6^	5.79 × 10^−6^	6.83 × 10^−6^	5.79 × 10^−6^	6.83 × 10^−6^
		Max	4.73 × 10^−3^	5.58 × 10^−3^	1.12 × 10^−5^	1.33 × 10^−4^	1.12 × 10^−2^	1.32 × 10^−2^
		Average	1.52 × 10^−5^	1.80 × 10^−5^	6.62 × 10^−6^	7.81 × 10^−6^	4.15 × 10^−5^	4.90 × 10^−5^
	Ciprofloxacin	Min	ND	ND	ND	ND	5.99 × 10^−5^	7.06 × 10^−5^
		Max	ND	ND	ND	ND	1.50 × 10^−3^	1.77 × 10^−3^
		Average	ND	ND	ND	ND	6.39 × 10^−5^	7.53 × 10^−4^
	Norfloxacin	Min	ND	ND	ND	ND	1.80 × 10^−6^	2.12 × 10^−6^
		Max	ND	ND	ND	ND	3.83 × 10^−5^	4.51 × 10^−5^
		Average	ND	ND	ND	ND	1.90 × 10^−6^	2.24 × 10^−6^
Sulfonamides	Sarafloxacin	Min	1.44 × 10^−7^	1.69 × 10^−7^	1.44 × 10^−7^	1.69 × 10^−7^	1.44 × 10^−7^	1.69 × 10^−7^
		Max	8.48 × 10^−6^	1.00 × 10^−5^	7.12 × 10^−4^	8.40 × 10^−4^	2.52 × 10^−3^	2.97 × 10^−3^
		Average	1.82 × 10^−7^	2.15 × 10^−7^	4.02 × 10^−6^	4.75 × 10^−6^	3.73 × 10^−6^	4.40 × 10^−6^
	Difluoroxacin	Min	1.44 × 10^−7^	1.69 × 10^−7^	1.44 × 10^−7^	1.69 × 10^−7^	ND	ND
		Max	4.23 × 10^−6^	4.99 × 10^−5^	5.34 × 10^−5^	6.31 × 10^−5^	ND	ND
		Average	2.05 × 10^−7^	2.42 × 10^−7^	2.96 × 10^−7^	3.49 × 10^−7^	ND	ND
	Ofloxacin	Min	7.18 × 10^−7^	8.47 × 10^−7^	7.18 × 10^−7^	8.47 × 10^−7^	ND	ND
		Max	6.56 × 10^−4^	7.73 × 10^−4^	5.24 × 10^−5^	6.19 × 10^−5^	ND	ND
		Average	2.18 × 10^−6^	2.57 × 10^−6^	8.02 × 10^−7^	9.46 × 10^−7^	ND	ND
	Sulfamonomethoxine	Min	1.44 × 10^−6^	1.69 × 10^−6^	1.44 × 10^−6^	1.69 × 10^−6^	1.44 × 10^−6^	1.69 × 10^−6^
		Max	9.42 × 10^−5^	1.11 × 10^−4^	3.02 × 10^−5^	3.56 × 10^−5^	1.61 × 10^−5^	1.90 × 10^−5^
		Average	1.77 × 10^−6^	2.08 × 10^−6^	1.61 × 10^−6^	1.90 × 10^−6^	1.47 × 10^−6^	1.73 × 10^−6^
	Sulfadimidine	Min	1.44 × 10^−6^	1.69 × 10^−6^	ND	ND	1.44 × 10^−6^	1.69 × 10^−6^
		Max	1.61 × 10^−3^	1.90 × 10^−3^	ND	ND	4.48 × 10^−5^	5.28 × 10^−5^
		Average	4.04 × 10^−6^	4.77 × 10^−6^	ND	ND	1.63 × 10^−6^	1.93 × 10^−6^
Macrolides	Sulfamethoxazole	Min	4.49 × 10^−6^	5.30 × 10^−6^	ND	ND	ND	ND
		Max	2.17 × 10^−4^	2.56 × 10^−4^	ND	ND	ND	ND
		Average	5.15 × 10^−6^	6.08 × 10^−6^	ND	ND	ND	ND
Tetracyclines	Sulfaquinoxaline	Min	2.39 × 10^−6^	2.82 × 10^−6^	ND	ND	2.39 × 10^−6^	2.82 × 10^−6^
		Max	2.67 × 10^−3^	3.15 × 10^−3^	ND	ND	2.91 × 10^−3^	3.43 × 10^−3^
		Average	6.45 × 10^−6^	7.61 × 10^−6^	ND	ND	1.43 × 10^−5^	1.69 × 10^−5^
Other	Sulfachloropyrazine sodium	Min	2.39 × 10^−7^	2.83 × 10^−7^	ND	ND	2.39 × 10^−7^	2.82 × 10^−7^
		Max	9.64 × 10^−5^	1.14 × 10^−4^	ND	ND	1.46 × 10^−4^	1.72 × 10^−4^
		Average	4.31 × 10^−7^	5.08 × 10^−7^	ND	ND	9.17 × 10^−7^	1.08 × 10^−6^
	Tilmicosin	Min	2.39 × 10^−6^	2.82 × 10^−6^	2.39 × 10^−6^	2.82 × 10^−6^	2.39 × 10^−6^	2.82 × 10^−6^
		Max	1.28 × 10^−2^	1.51 × 10^−2^	3.48 × 10^−2^	4.11 × 10^−2^	4.32 × 10^−2^	5.09 × 10^−2^
		Average	2.75 × 10^−4^	3.25 × 10^−4^	1.21 × 10^−4^	1.43 × 10^−4^	1.19 × 10^−4^	1.41 × 10^−4^

Note: ND means not detected.

**Table 7:** International Food Safety indices (IFS) of poultry eggs in Shandong, 2018–2020 (average intake of eggs, 50 g/d/person).

Antibiotics		Concentration (μg/kg)	2018		2019		2020	
			Male	Female	Male	Female	Male	Female
Fluoroquinolones	Enrofloxacin	Min	1.16 × 10^−5^	1.37 × 10^−5^	1.16 × 10^−5^	1.37 × 10^−5^	1.16 × 10^−5^	1.37 × 10^−5^
		Max	9.45 × 10^−3^	1.12 × 10^−2^	2.25 × 10^−4^	2.65 × 10^−4^	2.24 × 10^−2^	2.64 × 10^−2^
		Average	3.05 × 10^−5^	3.60 × 10^−5^	1.32 × 10^−5^	1.56 × 10^−5^	8.31 × 10^−6^	9.80 × 10^−5^
	Ciprofloxacin	Min	ND	ND	ND	ND	1.20 × 10^−4^	1.41 × 10^−4^
		Max	ND	ND	ND	ND	3.00 × 10^−3^	3.53 × 10^−3^
		Average	ND	ND	ND	ND	1.28 × 10^−4^	1.51 × 10^−4^
	Norfloxacin	Min	ND	ND	ND	ND	3.59 × 10^−6^	4.24 × 10^−6^
		Max	ND	ND	ND	ND	7.65 × 10^−5^	9.03 × 10^−5^
		Average	ND	ND	ND	ND	3.79 × 10^−6^	4.47 × 10^−6^
Sulfonamides	Sarafloxacin	Min	1.44 × 10^−7^	1.69 × 10^−7^	2.87 × 10^−7^	3.39 × 10^−7^	2.87 × 10^−7^	3.39 × 10^−7^
		Max	1.70 × 10^−5^	2.00 × 10^−5^	1.42 × 10^−3^	1.68 × 10^−3^	5.04 × 10^−3^	5.95 × 10^−3^
		Average	3.65 × 10^−7^	4.31 × 10^−7^	8.05 × 10^−6^	9.49 × 10^−6^	7.46 × 10^−6^	8.80 × 10^−6^
	Difluoroxacin	Min	1.44 × 10^−7^	1.69 × 10^−7^	2.87 × 10^−7^	3.39 × 10^−7^	ND	ND
		Max	8.46 × 10^−5^	9.98 × 10^−5^	1.07 × 10^−4^	1.26 × 10^−4^	ND	ND
		Average	4.09 × 10^−7^	4.83 × 10^−7^	5.92 × 10^−7^	6.98 × 10^−7^	ND	ND
	Ofloxacin	Min	7.18 × 10^−7^	8.47 × 10^−7^	1.44 × 10^−6^	1.69 × 10^−6^	ND	ND
		Max	1.31 × 10^−3^	1.55 × 10^−3^	1.05 × 10^−4^	1.24 × 10^−4^	ND	ND
		Average	4.36 × 10^−6^	5.14 × 10^−6^	1.60 × 10^−6^	1.89 × 10^−6^	ND	ND
	Sulfamonomethoxine	Min	1.44 × 10^−6^	1.69 × 10^−6^	2.87 × 10^−6^	3.39 × 10^−6^	2.87 × 10^−6^	3.39 × 10^−6^
		Max	1.88 × 10^−4^	2.22 × 10^−4^	6.03 × 10^−5^	7.12 × 10^−5^	3.21 × 10^−5^	3.80 × 10^−5^
		Average	3.53 × 10^−6^	4.17 × 10^−6^	3.22 × 10^−6^	3.80 × 10^−6^	2.94 × 10^−6^	3.47 × 10^−6^
	Sulfadimidine	Min	1.44 × 10^−6^	1.69 × 10^−6^	ND	ND	2.87 × 10^−6^	3.39 × 10^−6^
		Max	3.22 × 10^−3^	3.80 × 10^−3^	ND	ND	8.95 × 10^−5^	1.06 × 10^−4^
		Average	8.08 × 10^−6^	9.53 × 10^−6^	ND	ND	3.27 × 10^−6^	3.86 × 10^−6^
Macrolides	Sulfamethoxazole	Min	4.49 × 10^−6^	5.30 × 10^−6^	ND	ND	ND	ND
		Max	4.35 × 10^−4^	5.13 × 10^−4^	ND	ND	ND	ND
		Average	1.03 × 10^−5^	1.22 × 10^−5^	ND	ND	ND	ND
Tetracyclines	Sulfaquinoxaline	Min	2.39 × 10^−6^	2.82 × 10^−6^	ND	ND	4.79 × 10^−6^	5.65 × 10^−6^
		Max	5.34 × 10^−3^	6.30 × 10^−3^	ND	ND	5.82 × 10^−3^	6.86 × 10^−3^
		Average	1.29 × 10^−5^	1.52 × 10^−5^	ND	ND	2.86 × 10^−5^	3.37 × 10^−5^
Other	Sulfachloropyrazine sodium	Min	2.39 × 10^−7^	2.83 × 10^−7^	ND	ND	4.79 × 10^−7^	5.65 × 10^−7^
		Max	1.93 × 10^−4^	2.27 × 10^−4^	ND	ND	2.92 × 10^−4^	3.45 × 10^−4^
		Average	8.62 × 10^−7^	1.02 × 10^−6^	ND	ND	1.83 × 10^−6^	2.15 × 10^−6^
	Tilmicosin	Min	2.39 × 10^−6^	2.82 × 10^−6^	4.79 × 10^−6^	5.65 × 10^−6^	4.79 × 10^−6^	5.65 × 10^−6^
		Max	2.56 × 10^−2^	3.02 × 10^−2^	6.96 × 10^−2^	8.21 × 10^−2^	8.64 × 10^−2^	0.102
		Average	5.51 × 10^−4^	6.49 × 10^−4^	2.42 × 10^−4^	2.86 × 10^−4^	2.38 × 10^−4^	2.81 × 10^−4^

Note: ND means not detected.

## Data Availability

With regard to the data presented in this study, we included them in the article.

## Supplementary Materials

The following are available online at https://www.mdpi.com/article/10.3390/vetsci9030126/s1, File S1: Extraction and analytical method, validation process for the analysis of the eggs in the UPLC-MS spectrometry.
