# Supplementary material for: Risk Analysis of 24 Residual Antibiotics in Poultry Eggs in Shandong, China (2018–2020)
Source: Vet Sci. 2022 Mar 10;9(3):126. doi: 10.3390/vetsci9030126 (PMC8953159; doi:10.3390/vetsci9030126)
Supplement: Supplementary file 1 [file vetsci-09-00126-s001.zip › vetsci-1501024-Supplementary-File S1.pdf]

Extraction and Analytical Method, Validation Process for the Analysis of Eggs in the UPLC-MS Spectrometry

Highlight: A method based on ultra-performance liquid chromatography coupled with tandem mass spectrometry (UPLC-MS/MS) was developed for the simultaneous determination of 24 antibiotic residues in eggs. This method was based on the new solid-phase extraction column, PRIME HLB. The targeted compounds in eggs were extracted with acetonitrile / water (80:20, V/V) containing 0.02 mol/L Ethylene Diamine Tetraacetic Acid (EDTA), then through PRIME HLB, and the matrix-matching external standard method was used for quantification. The average recoveries of the 24 analytes ranged from 71.4% to 108.2%, and the relative standard deviation (RSD) in three different concentrations was 0.7–8.1%. The limit of detection (LOD,S/N ≥ 3) and quantification (LOQ, S/N ≥ 10) were 0.01-1.0 μg/kg and 0.05–3.5 μg/kg, respectively. The method is simple, fast, sensitive and reliable, and it is suitable for a rapid determination of drug residues in eggs.

Materials and Methods

Instruments

Ultra-performance liquid chromatography–tandem mass spectrometry (Waters xevo TQ-S, Shanghai woteshi Technology Co., Ltd., China), equipped with electrospray ionization (ESI) and Masslynx data processing system; electronic balance (BT125D, Germany Sartorius Company, Germany); desktop high-speed freezing centrifuge (Allegra64R, Beckman company, USA); vortex mixer (XW-80A, Shanghai Jingke Industrial Co., LTD., China); vertical multi-purpose vibrator (Hy-4, Beijing Tajin Technology Co., LTD., China); ultra-pure water system (Milli PAK, Millpore, USA); solid-phase extraction column (Oasis PRiME HLB, Waters company, USA).

Reagents

Enrofloxacin(China Veterinary Drug Control Institute, Batch No. H0081206, China);
Sarafloxacin(China Veterinary Drug Control Institute, Batch No. H0181108, China);
Darfloxacin(China Veterinary Drug Control Institute, Batch No. H0201210, China);
Norfloxacin(China Veterinary Drug Control Institute, Batch No.H0071305, China);
Lomefloxacin(China Veterinary Drug Control Institute, Batch No. H0121505, China);
Sulfamonomethoxine(China Veterinary Drug Control Institute, Batch No. C0031610, China);
Sulfadimidine(China Veterinary Drug Control Institute, Batch No. C0061007, China);
Sulfamethoxazole(China Veterinary Drug Control Institute, Batch No. H0261106, China);
Sulfadimethoxine(China Veterinary Drug Control Institute, Batch No. H0371406, China);
Sulfaquinoxaline(China Veterinary Drug Control Institute, Batch No. H0251407, China);
Sulfachloropyrazine sodium(Institute of Environmental Protection, Ministry of Agriculture, Batch No. SB06-087-2008, China);
Sulfaclodazine sodium(Institute of Environmental Protection, Ministry of Agriculture, Batch No. SB05-083-2008, China);
Amantadine(Shanghai AMp Scientific Instrument Co., LTD., Batch No. A0309578, China);
Rimantadine(Shanghai AMp Scientific Instrument Co., LTD., Batch No. A0304758, China);

Tylosin(China Veterinary Drug Control Institute, Batch No. K0161305, China);
Tilmicosin(China Veterinary Drug Control Institute, Batch No. K0311407, China);
Erythromycin(China Veterinary Drug Control Institute, Batch No. K40528, China);
Azithromycin(China National Institute for Food and Drug Control, Batch No. 130593-201303, China);
Oxytetracycline(China Veterinary Drug Control Institute, Batch No. K0031208, China);
Tetracycline(China Veterinary Drug Control Institute, Batch No. 0011209, China);
Doxycycline(China Veterinary Drug Control Institute, Batch No. 0131209, China);
Aureomycin(China Veterinary Drug Control Institute, Batch No. 0041110, China);
Lincomycin (China Veterinary Drug Control Institute, Batch No. 0101503, China);
Florfenicol(China Veterinary Drug Control Institute, Batch No. K0320906, China);
Acetonitrile(chromatography pure, Merck company, Germany); methanol (chromatography pure, Merck company, Germany); formic acid (chromatographic pure, Sigma-Aldrich Company, USA); deionized water (Milli PAK ultra-pure water system, Millpore, USA); Ethylene Diamine Tetraacetic Acid, EDTA (analytical pure, Shanghai Sinopharm Chemical Reagents Co., LTD.,China).

Standard Stock Solutions

An amount of 0.01g (accurate to 0.1mg) of each antibiotic reagent was carefully weighed and placed in a brown volumetric flask of 10 ml, and then the volume was fixed with methanol. The concentration of standard stock solution was 1 mg/mL, and it was stored in the refrigerator at -20 °C.

Mixed Standard Working Solutions

Standard stock solutions were diluted to become mixed standard working solutions. The concentration of mixed standard working solution was 10 mg /L.

Sample Preparation and Extraction Procedure

First, 2.00 ± 0.02 g of mixed egg liquid was weighed into a 50 mL centrifuge tube and spiked with 2 mL of 0.02 mol/L EDTA solution. After 1 minute of vortexing, 8 mL of acetonitrile was added to the solution. Then, the solution was subjected to vortices for 1 min, ultrasonic extraction for 10 min and high-speed centrifugation at 5000 r/min for 5 min. Without activation and balance, 5 mL of supernatant from the treated solution was directly loaded into a 6cc PRiME HLB solid-phase extraction column, and all effluent from the column was collected. After the effluent was blown to nearly dry with nitrogen at 40 °C, it was constant volume at 1.00 mL with 20% methanol aqueous solution. Finally, the solution was filtered with 0.2 μm microporous membrane.

UPLC-MS/MS Analysis

Chromatographic column: BEH C18 (100 mm × 2.1 mm, 1.7 μm)； flow rate: 0.2 ml/min; injection volume: 1 μL; column temperature: 35 °C.

Mobile phase: in positive ion mode, mobile phase A is methanol, and mobile phase B is 0.1% formic acid water; in negative ion mode, mobile phase A is methanol, and mobile phase B is water.

The HPLC gradient elution program is shown in Table S1.

Mass Spectrometry Conditions

Electrospray ionization source; multiple response monitoring (MRM); ion source temperature: 150 °C; solvent removal temperature: 400 °C; flow rate of dissolvent-nitrogen: 900 L/h; capillary voltage: 3 kV.

The mother ion, sub ion, taper hole voltage and collision are shown in Table S2.

Table S1. HPLC gradient elution program.

Time/min	A/%	B/%	Curve
0	20	80	Initial condition
2.0	70	30	6
3.5	90	10	6
4.5	20	80	6
6	20	80	1

Table S2. Table of MRM ion monitor parameters of the 24 antibiotics.

Antibiotics	Mother ion	Sub ion	Taper hole voltage/V	Collision energy/eV	Ion source
Enrofloxacin	360.12	245.13	52	26	ESI^+
		316.15	52	18	ESI^+
Sarafloxacin	386.10	299.09	46	26	ESI^+
		368.12	46	21	ESI^+
Darfloxacin	358.13	255.07	36	17	ESI^+
		340.14	36	35	ESI^+
Norfloxacin	320.15	233.26	42	24	ESI^+
		302.29	42	19	ESI^+
Lomefloxacin	352.30	265.20	36	23	ESI^+
		308.30	36	17	ESI^+
Sulfamonomethoxine	281.10	126.10	18	19	ESI^+
		156.10	18	16	ESI^+
Sulfadimidine	279.20	124.20	20	24	ESI^+
		186.20	20	16	ESI^+
Sulfamethoxazole	254.10	108.10	22	22	ESI^+
		156.10	22	16	ESI^+
Sulfadimethoxine	311.20	108.10	20	30	ESI^+
		156.140	20	18	ESI^+
Sulfaquinoxaline	301.10	108.10	30	16	ESI^+
		156.10	30	30	ESI^+
Sulfachloropyrazine sodium	284.90	108.00	19	35	ESI^+
		108.10	19	35	ESI^+
Sulfaclodazine sodium	284.80	107.90	24	24	ESI^+

		155.80	24	13	ESI^+
Amantadine	152.00	92.90	30	25	ESI^+
		135.00	30	15	ESI^+
Rimantadine	187.12	106.13	30	24	ESI^+
		110.1	30	24	ESI^+
Tylosin	916.50	145.00	20	40	ESI^+
		174.10	20	30	ESI^+
Tilmicosin	869.80	174.00	20	40	ESI^+
		696.50	20	40	ESI^+
Erythromycin	734.50	158.10	30	30	ESI^+
		576.50	30	20	ESI^+
Azithromycin	844.12	159.13	20	40	ESI^+
		177.00	20	20	ESI^+
Oxytetracycline	461.30	426.40	30	15	ESI^+
		443.40	30	10	ESI^+
Tetracycline	445.40	154.20	35	20	ESI^+
		410.40	35	15	ESI^+
Doxycycline	445.40	154.10	30	25	ESI^+
		428.40	30	15	ESI^+
Aureomycin	479.50	154.10	30	20	ESI^+
		444.40	30	15	ESI^+
Lincomycin	407.20	126.00	20	25	ESI^+
		359.10	20	18	ESI^+
Florfenicol	358.00	184.83	30	20	ESI^+
		337.96	30	8	ESI^+

Note: ESI means electrospray ionization.

Results

Recovery, Precision, Limit of Detection and Limit of Quantification of the Method

In order to reduce the matrix effect, blank matrix-matched standard solution was used for quantification, and egg extract was used as matrix background solution. Under the above optimized conditions, the mixed standard solution was accurately added into the blank egg sample, and the matrix-matched standard sample was used for quantification. The egg samples were tested by adding standard substance with three concentrations of limit of quantification (LOQ), 2 times limit of quantification (2×) and 5 times limit of quantification (5×). Each concentration was set in six parallels.

The results of limit of detection (LODs), limit of quantification (LOQs), recoveries (%) and relative standard deviation (RSDs) of 24 antibiotics are shown in Table S3. The average recoveries of the 24 analytes ranged from 71.4% to 108.2%, and the relative standard deviation (RSD) in three different concentrations was 0.7–8.1%. The limits of detection (LOD, $S/N \geq 3$) and quantification (LOQ, $S/N \geq 10$) were 0.01-1.0 μg/kg and 0.05–3.5 μg/kg, respectively.

Table S3. LODs，LOQs，average recoveries and RSDs of the 24 antibiotics in eggs (*n* = 6).

Antibiotics	LODs μg/kg	LOQs μg/kg	LOQs		2 times LOQs		5 times LOQs	
			Recovery rate/%	RSD/%	Recovery rate/%	RSD/%	Recovery rate/%	RSD/%
Enrofloxacin	0.1	0.4	79.4	5.6	77.4	3.4	82.4	4.7
Sarafloxacin	0.5	2.5	77.7	3.1	72.3	2.5	74.3	1.9
Darfloxacin	0.1	0.4	88.4	2.9	79.7	3.5	85.4	2.0
Norfloxacin	0.5	2.0	87.8	4.9	79.6	2.3	83.2	1.7
Lomefloxacin	0.02	0.07	84.5	5.1	81.7	2.2	84.4	2.5
Sulfamonomethoxine	0.02	0.1	89.1	2.2	84.3	3.1	85.7	2.7
Sulfadimidine	0.02	0.1	77.7	2.1	75.4	2.0	76.0	1.9
Sulfamethoxazole	0.1	0.3	84.5	2.7	83.7	2.1	82.9	1.7
Sulfadimethoxine	0.02	0.1	78.3	1.9	74.9	2.0	75.1	1.4
Sulfaquinoxaline	0.2	0.8	81.9	4.9	83.2	3.1	79.8	1.5
Sulfachloropyrazine sodium	0.2	0.8	83.2	3.9	84.1	2.9	80.2	0.7
Sulfaclodazine sodium	0.02	0.1	79.3	5.9	85.2	3.2	77.9	1.6
Amantadine	0.5	2.5	88.7	3.7	90.4	2.1	89.1	1.9
Rimantadine	0.5	2.0	80.1	4.5	88.0	2.1	85.1	2.4
Tylosin	0.2	0.8	101.1	7.7	105.7	5.8	99.4	5.9
Tilmicosin	0.5	2.0	108.2	8.1	104.1	5.9	95.7	6.1
Erythromycin	0.02	0.07	89.7	8.0	82.4	5.4	84.3	4.7
Azithromycin	0.5	2.2	106.2	8.8	100.3	5.5	96.8	5.4
Oxytetracycline	0.02	0.08	75.7	2.9	77.9	3.7	74.5	2.1
Tetracycline	0.02	0.08	71.4	4.2	79.4	3.7	77.8	2.0
Doxycycline	0.02	0.08	73.3	3.0	71.3	2.2	73.4	2.4
Aureomycin	0.2	0.7	77.5	3.1	74.4	2.1	72.1	0.7
Lincomycin	0.02	0.08	107.1	2.4	97.2	3.4	93.1	2.0
Florfenicol	0.02	0.07	82.9	3.9	81.6	3.1	87.5	1.4

Note: LOD means limit of detection; LOQ means limit of quantification; RSD means relative standard deviation.

Determination of Actual Samples (Validation)

The method was used to detect 200 egg samples (collected from supermarkets and farmers' markets in Shandong), and nine egg samples of antibiotic residues were detected, including sulfamonomethoxine (one egg, 0.57 μg/kg), sulfamethoxazole (one egg, 1.20 μg/kg), doxycycline (two eggs, 4.33, 22.31 μg/kg), tilmicosin (two eggs, 8.93, 6.46 μg/kg), norfloxacin (one egg, 7.53 μg/kg) and florfenicol (two eggs, 1.0, 11.2 μg/kg). The nine samples were re-examined by ISO method, and the results were consistent, which further proved the accuracy of the method. The multi-reaction monitoring (MRM) chromatograms of typical samples are shown in Figure S1.

Sulfamonomethoxine:

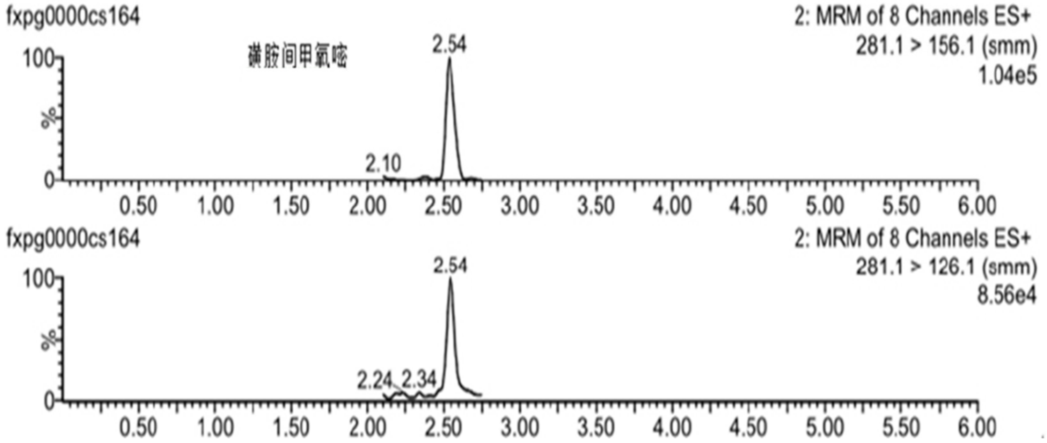

Sulfamethoxazole:

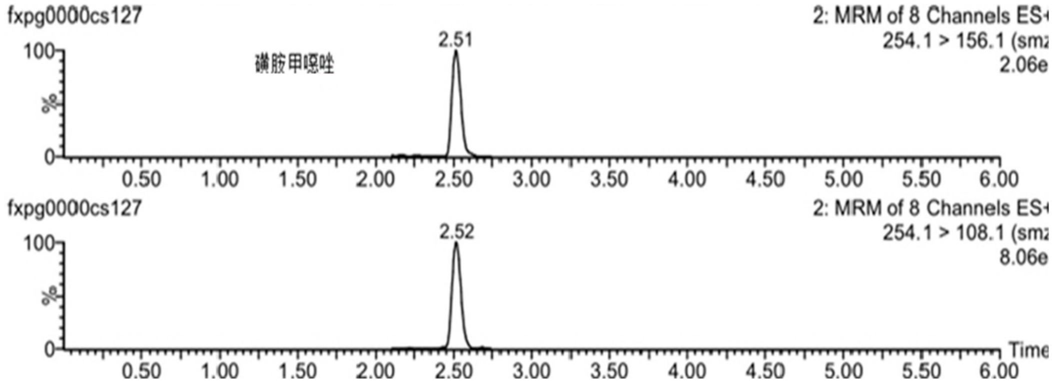

Doxycycline:

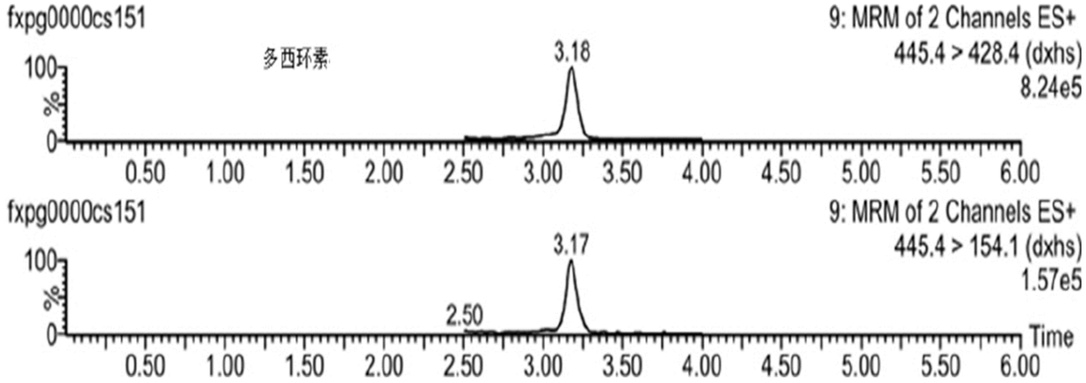

Tilmicosin:

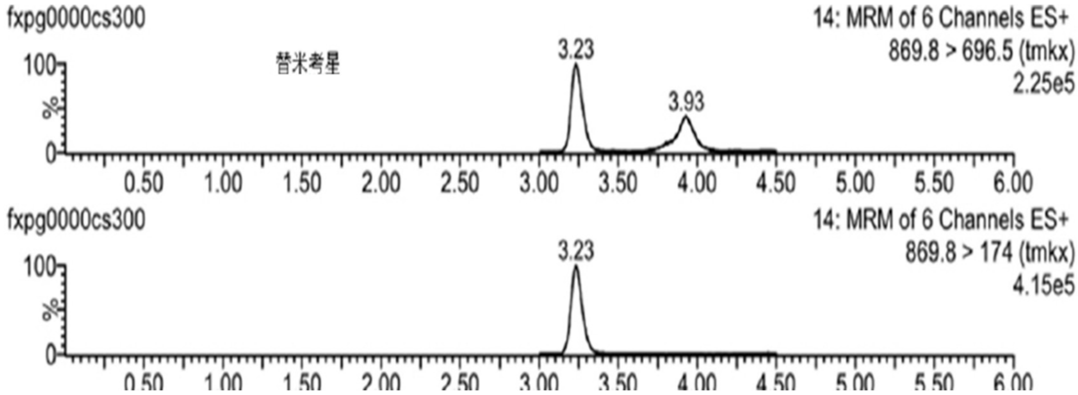

Norfloxacin:

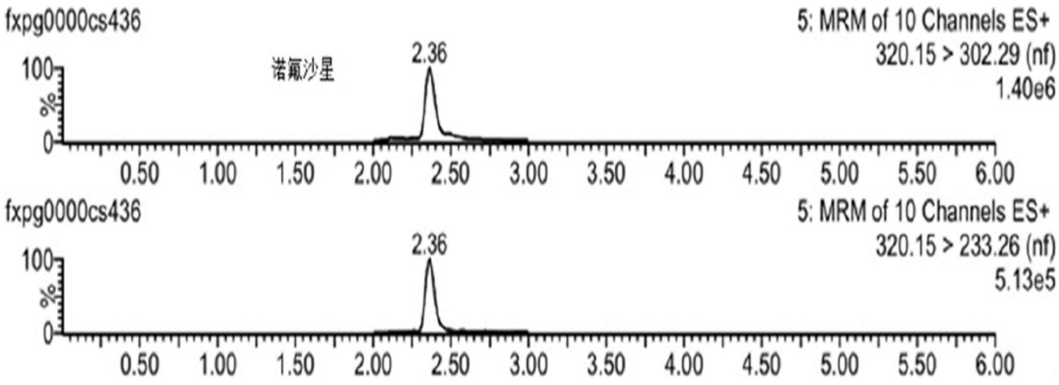

Florfenicol:

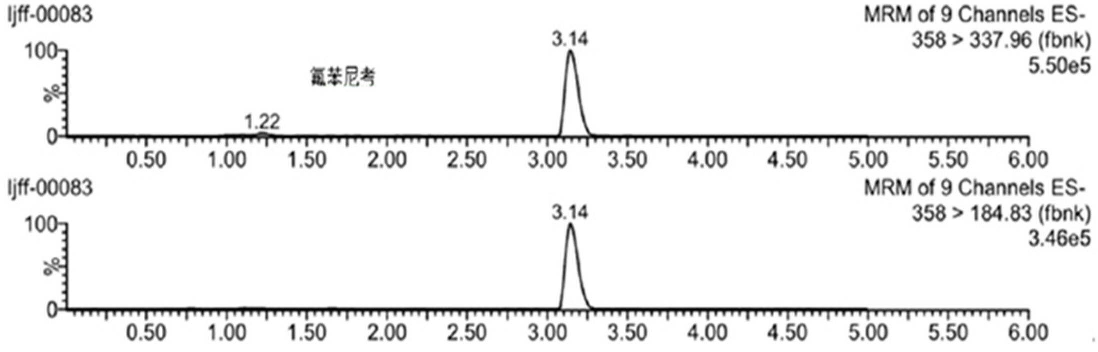

Figure S1. MRM chromatogram of typical samples.
